# Supplementary material for: Computational design of novel nanobodies targeting the receptor binding domain of variants of concern of SARS-CoV-2
Source: PLoS One. 2023 Oct 24;18(10):e0293263. doi: 10.1371/journal.pone.0293263 (PMC10597523; doi:10.1371/journal.pone.0293263)
Supplement: S1 Table — (PDF) [file pone.0293263.s001.pdf]

S1 Table.

No.	PDB ID	Structure investigation method	Resolution (Å)	RBD		Ligand (Nb or Ab fragment)	
				RBD variant	RBD chain	Source	Nb chain
1.1	6YZ5	X-RAY DIFFRACTION	1.8	Wh	A	Lama glama, Nanobody	B
2.1	6YZ7	X-RAY DIFFRACTION	3.3	Wh	A	Lama glama, Nanobody	D
3.1	6Z2M	X-RAY DIFFRACTION	2.71	Wh	A	Lama glama, Nanobody	H
4.1	6ZBP	X-RAY DIFFRACTION	1.85	Wh	A	Lama glama, Nanobody	B
5.1	6ZCZ	X-RAY DIFFRACTION	2.65	Wh	A	Lama glama, Nanobody	B
6.1	6ZH9	X-RAY DIFFRACTION	3.31	Wh	C	Lama glama, Nanobody	D
7.1	6ZHD	ELECTRON MICROSCOPY	3.7	Wh	A	Lama glama, Nanobody	D
8.1	6ZXN	ELECTRON MICROSCOPY	2.93	Wh	A	Vicugna pacos, Nanobody	D
9.1	7A25	ELECTRON MICROSCOPY	3.06	Wh	A	Synthetic Nanobody	D
10.1	7A29	ELECTRON MICROSCOPY	2.94	Wh	A	Synthetic Nanobody	D
11.1	7B14	ELECTRON MICROSCOPY	3.79	Wh	A	Camelus bactrianus, Nanobody	B
12.1	7B17	X-RAY DIFFRACTION	4.01	Wh	A	Lama glama, Nanobody	B1
12.2	7B17	X-RAY DIFFRACTION	4.01	Wh	A	Lama glama, Nanobody	B2
13.1	7B18	ELECTRON MICROSCOPY	2.62	Wh	A	Lama glama, Nanobody	D
14.1	7C8V	X-RAY DIFFRACTION	2.15	Wh	B	Synthetic Nanobody	A
15.1	7JVB	X-RAY DIFFRACTION	3.29	Wh	A	Lama glama, Nanobody	B
16.1	7KGJ	X-RAY DIFFRACTION	2.3	Wh	A	Synthetic Nanobody	B
17.1	7KGK	X-RAY DIFFRACTION	2.6	Wh	A	Synthetic Nanobody	B
18.1	7KKK	ELECTRON MICROSCOPY	3.03	Wh	A	Synthetic Nanobody	B
19.1	7KKL	ELECTRON MICROSCOPY	2.85	Wh	A	Synthetic Nanobody	B
20.1	7KLW	X-RAY DIFFRACTION	2.6	Wh	A	Synthetic Nanobody	B
20.2	7KLW	X-RAY DIFFRACTION	2.6	Wh	A	Synthetic Nanobody	C
21.1	7KN5	X-RAY DIFFRACTION	1.87	Wh	A	Vicugna pacos, Nanobody	C
21.2	7KN5	X-RAY DIFFRACTION	1.87	Wh	A	Vicugna pacos, Nanobody	E
22.1	7LX5	X-RAY DIFFRACTION	3.44	Wh	A	Vicugna pacos, Nanobody	B
22.2	7LX5	X-RAY DIFFRACTION	3.44	Wh	A	Vicugna pacos, Nanobody	C
23.1	7MFU	X-RAY DIFFRACTION	1.7	Wh	A	Synthetic Nanobody	B
23.2	7MFU	X-RAY DIFFRACTION	1.7	Wh	A	Synthetic Nanobody	C
24.1	7NKT	X-RAY DIFFRACTION	2.3	Wh	A	Vicugna pacos, Nanobody	B
25.1	6Z2M	X-RAY DIFFRACTION	2.71	Wh	A	Homo sapiens, Ab (heavy chain)	B
						Homo sapiens, Ab (Light chain)	C
26.1	6XC2	X-RAY DIFFRACTION	3.11	Wh	A	Homo sapiens, Ab (heavy chain)	C
						Homo sapiens, Ab (Light chain)	B
27.1	6XC3	X-RAY DIFFRACTION	2.7	Wh	E	Homo sapiens, Ab (heavy chain)	D
						Homo sapiens, Ab (Light chain)	C
28.1	6XC4	X-RAY DIFFRACTION	2.34	Wh	A	Homo sapiens, Ab (heavy chain)	B
						Homo sapiens, Ab (Light chain)	C
29.1	6XC7	X-RAY DIFFRACTION	2.88	Wh	A	Homo sapiens, Ab (heavy chain)	B

							Homo sapiens, Ab (Light chain)	C
30.1	6XKP	X-RAY DIFFRACTION	2.78	Wh	A	Homo sapiens, Ab (heavy chain)	C
							Homo sapiens, Ab (Light chain)	D
31.1	6YM0	X-RAY DIFFRACTION	4.36	Wh	A	Homo sapiens, Ab (heavy chain)	B
							Homo sapiens, Ab (Light chain)	C
32.1	6YOR	ELECTRON MICROSCOPY	3.3	Wh	A	Homo sapiens, Ab (heavy chain)	B
							Homo sapiens, Ab (Light chain)	C
33.1	6YZ7	X-RAY DIFFRACTION	3.3	Wh	A	Homo sapiens, Ab (heavy chain)	B
							Homo sapiens, Ab (Light chain)	C
34.1	6ZCZ	X-RAY DIFFRACTION	2.65	Wh	A	Homo sapiens, Ab (heavy chain)	C
							Homo sapiens, Ab (Light chain)	D
35.1	6ZFO	ELECTRON MICROSCOPY	4.4	Wh	A	Homo sapiens, Ab (heavy chain)	B
							Homo sapiens, Ab (Light chain)	C
36.1	6ZH9	X-RAY DIFFRACTION	3.31	Wh	C	Homo sapiens, Ab (heavy chain)	A
							Homo sapiens, Ab (Light chain)	B
37.1	7B3O	X-RAY DIFFRACTION	2	Wh	A	Homo sapiens, Ab (heavy chain)	C
							Homo sapiens, Ab (Light chain)	B
38.1	7BEH	X-RAY DIFFRACTION	2.3	Wh	**A**	Homo sapiens, Ab (heavy chain)	B
							Homo sapiens, Ab (Light chain)	A
39.1	7BEI	X-RAY DIFFRACTION	2.3	Wh	**C**	Homo sapiens, Ab (heavy chain)	A
							Homo sapiens, Ab (Light chain)	B
40.1	7BEJ	X-RAY DIFFRACTION	2.42	Wh	**C**	Homo sapiens, Ab (heavy chain)	A
							Homo sapiens, Ab (Light chain)	B
41.1	7BEK	X-RAY DIFFRACTION	2.04	Wh	C	Homo sapiens, Ab (heavy chain)	A
							Homo sapiens, Ab (Light chain)	B
42.1	7BEL	X-RAY DIFFRACTION	2.53	Wh	**A**	Homo sapiens, Ab (heavy chain)	**C**
							Homo sapiens, Ab (Light chain)	**D**
42.2	7BEL	X-RAY DIFFRACTION	2.53	Wh	A	Homo sapiens, Ab (heavy chain)	B
							Homo sapiens, Ab (Light chain)	E
43.1	7BEM	X-RAY DIFFRACTION	2.52	Wh	C	Homo sapiens, Ab (heavy chain)	A
							Homo sapiens, Ab (Light chain)	B
44.1	7BEO	X-RAY DIFFRACTION	3.19	Wh	A	Homo sapiens, Ab (heavy chain)	B
							Homo sapiens, Ab (Light chain)	C
45.1	7C01	X-RAY DIFFRACTION	2.88	Wh	A	Homo sapiens, Ab (heavy chain)	B
							Homo sapiens, Ab (Light chain)	C
46.1	7CAC	ELECTRON MICROSCOPY	3.55	Wh	A	Homo sapiens, Ab (heavy chain)	E
							Homo sapiens, Ab (Light chain)	D
47.1	7CAI	ELECTRON MICROSCOPY	3.49	Wh	A	Homo sapiens, Ab (heavy chain)	E
							Homo sapiens, Ab (Light chain)	D
48.1	7CAK	ELECTRON MICROSCOPY	3.58	Wh	A	Homo sapiens, Ab (heavy chain)	E
							Homo sapiens, Ab (Light chain)	D

49.1	7CHB	X-RAY DIFFRACTION	2.4	Wh	C	Homo sapiens, Ab (heavy chain)	C
						Homo sapiens, Ab (Light chain)	B
50.1	7CJF	X-RAY DIFFRACTION	2.11	Wh	C	Homo sapiens, Ab (heavy chain)	A
						Homo sapiens, Ab (Light chain)	B
51.1	7CM4	X-RAY DIFFRACTION	2.71	Wh	A	Homo sapiens, Ab (heavy chain)	B
						Homo sapiens, Ab (Light chain)	C
52.1	7CWN	ELECTRON MICROSCOPY	3.2	Wh	A	Homo sapiens, Ab (heavy chain)	L
						Homo sapiens, Ab (Light chain)	M
53.1	7CZQ	ELECTRON MICROSCOPY	2.8	Wh	A	Homo sapiens, Ab (heavy chain)	D
						Homo sapiens, Ab (Light chain)	E
54.1	7CZT	ELECTRON MICROSCOPY	2.7	Wh	A	Homo sapiens, Ab (heavy chain)	D
						Homo sapiens, Ab (Light chain)	E
55.1	7CZZ	ELECTRON MICROSCOPY	3.2	Wh	A	Homo sapiens, Ab (heavy chain)	D
						Homo sapiens, Ab (Light chain)	E
56.1	7D00	ELECTRON MICROSCOPY	3	Wh	B	Homo sapiens, Ab (heavy chain)	D
						Homo sapiens, Ab (Light chain)	E
57.1	7DD2	ELECTRON MICROSCOPY	5.6	Wh	A	Homo sapiens, Ab (heavy chain)	F
						Homo sapiens, Ab (Light chain)	G
58.1	7DD8	ELECTRON MICROSCOPY	7.5	Wh	C	Homo sapiens, Ab (heavy chain)	A
						Homo sapiens, Ab (Light chain)	B
59.1	7DEO	X-RAY DIFFRACTION	2.5	Wh	A	Homo sapiens, Ab (heavy chain)	D
						Homo sapiens, Ab (Light chain)	E
60.1	7DET	X-RAY DIFFRACTION	2.2	Wh	B	Homo sapiens, Ab (heavy chain)	B
						Homo sapiens, Ab (Light chain)	B
61.1	7DK4	ELECTRON MICROSCOPY	3.8	Wh	C	Homo sapiens, Ab (heavy chain)	A
						Homo sapiens, Ab (Light chain)	B
62.1	7DK5	ELECTRON MICROSCOPY	13.5	Wh	A	Homo sapiens, Ab (heavy chain)	C
						Homo sapiens, Ab (Light chain)	D
63.1	7DK6	ELECTRON MICROSCOPY	4.3	Wh	A	Homo sapiens, Ab (heavy chain)	D
						Homo sapiens, Ab (Light chain)	E
64.1	7DK7	ELECTRON MICROSCOPY	9.7	Wh	C	Homo sapiens, Ab (heavy chain)	A
						Homo sapiens, Ab (Light chain)	B
65.1	7DPM	X-RAY DIFFRACTION	3.3	Wh	A	Homo sapiens, Ab (heavy chain)	C
						Homo sapiens, Ab (Light chain)	B
66.1	7E23	ELECTRON MICROSCOPY	3.3	Wh	A	Homo sapiens, Ab (heavy chain)	B
						Homo sapiens, Ab (Light chain)	C
67.1	7E86	X-RAY DIFFRACTION	2.9	Wh	A	Homo sapiens, Ab (heavy chain)	B
						Homo sapiens, Ab (Light chain)	C
68.1	7E88	X-RAY DIFFRACTION	3.14	Wh	C	Homo sapiens, Ab (heavy chain)	A
						Homo sapiens, Ab (Light chain)	B
69.1	7JMO	X-RAY DIFFRACTION	2.36	Wh	A	Homo sapiens, Ab (heavy chain)	B
						Homo sapiens, Ab (Light chain)	C

70.1	7JVA	ELECTRON MICROSCOPY	3.6	Wh	C	Homo sapiens, Ab (heavy chain)	A
						Homo sapiens, Ab (Light chain)	B
71.1	7JX3	X-RAY DIFFRACTION	2.65	Wh	G	Homo sapiens, Ab (heavy chain)	D
						Homo sapiens, Ab (Light chain)	C
71.2	7JX3	X-RAY DIFFRACTION	2.65	Wh	G	Homo sapiens, Ab (heavy chain)	B
						Homo sapiens, Ab (Light chain)	A
71.3	7JX3	X-RAY DIFFRACTION	2.65	Wh	G	Homo sapiens, Ab (heavy chain)	L
						Homo sapiens, Ab (Light chain)	H
72.1	7K8M	X-RAY DIFFRACTION	3.2	Wh	C	Homo sapiens, Ab (heavy chain)	A
						Homo sapiens, Ab (Light chain)	B
73.1	7K8S	ELECTRON MICROSCOPY	3.4	Wh	A	Homo sapiens, Ab (heavy chain)	D
						Homo sapiens, Ab (Light chain)	E
74.1	7K8T	ELECTRON MICROSCOPY	3.4	Wh	A	Homo sapiens, Ab (heavy chain)	H
						Homo sapiens, Ab (Light chain)	I
75.1	7K8X	ELECTRON MICROSCOPY	3.9	Wh	A	Homo sapiens, Ab (heavy chain)	D
						Homo sapiens, Ab (Light chain)	G
76.1	7KFV	X-RAY DIFFRACTION	2.1	Wh	A	Homo sapiens, Ab (heavy chain)	H
						Homo sapiens, Ab (Light chain)	I
77.1	7KFW	X-RAY DIFFRACTION	2.79	Wh	A	Homo sapiens, Ab (heavy chain)	H
						Homo sapiens, Ab (Light chain)	I
78.1	7KFX	X-RAY DIFFRACTION	2.23	Wh	A	Homo sapiens, Ab (heavy chain)	B
						Homo sapiens, Ab (Light chain)	C
79.1	7KMG	X-RAY DIFFRACTION	2.16	Wh	C	Homo sapiens, Ab (heavy chain)	A
						Homo sapiens, Ab (Light chain)	B
80.1	7KML	ELECTRON MICROSCOPY	3.8	Wh	A	Homo sapiens, Ab (heavy chain)	E
						Homo sapiens, Ab (Light chain)	D
81.1	7KQB	ELECTRON MICROSCOPY	2.42	Wh	A	Homo sapiens, Ab (heavy chain)	D
						Homo sapiens, Ab (Light chain)	E
82.1	7KS9	ELECTRON MICROSCOPY	4.75	Wh	D	Homo sapiens, Ab (heavy chain)	B
						Homo sapiens, Ab (Light chain)	A
83.1	7LJR	ELECTRON MICROSCOPY	3.66	Wh	A	Homo sapiens, Ab (heavy chain)	D
						Homo sapiens, Ab (Light chain)	E
84.1	7M3I	X-RAY DIFFRACTION	2.8	Wh	C	Homo sapiens, Ab (heavy chain)	A
						Homo sapiens, Ab (Light chain)	B
85.1	7M6D	X-RAY DIFFRACTION	3.1	Wh	C	Homo sapiens, Ab (heavy chain)	A
						Homo sapiens, Ab (Light chain)	B
85.2	7M6D	X-RAY DIFFRACTION	3.1	Wh	C	Homo sapiens, Ab (heavy chain)	D
						Homo sapiens, Ab (Light chain)	E
86.1	7M6E	ELECTRON MICROSCOPY	3.3	Wh	A	Homo sapiens, Ab (heavy chain)	F
						Homo sapiens, Ab (Light chain)	I
87.1	7M6H	ELECTRON MICROSCOPY	4	Wh	A	Homo sapiens, Ab (heavy chain)	E

						Homo sapiens, Ab (Light chain)	G
88.2	7M7W	X-RAY DIFFRACTION	2.65	Wh	I	Homo sapiens, Ab (heavy chain)	B
						Homo sapiens, Ab (Light chain)	A
88.3	7M7W	X-RAY DIFFRACTION	2.65	Wh	I	Homo sapiens, Ab (heavy chain)	D
						Homo sapiens, Ab (Light chain)	C
89.1	7MJH	ELECTRON MICROSCOPY	2.66	Alpha (N501Y)	A	Synthetic, Ab (heavy chain)	D
90.1	7MJI	ELECTRON MICROSCOPY	2.81	Alpha (N501Y)	A	Synthetic, Ab (heavy chain)	B
91.1	7MJJ	ELECTRON MICROSCOPY	3.32	Alpha (N501Y)	A	Synthetic, Ab (heavy chain)	D
						Synthetic, Ab (Light chain)	E
92.1	7MJK	ELECTRON MICROSCOPY	2.73	Alpha (N501Y)	A	Synthetic, Ab (heavy chain)	B
						Synthetic, Ab (Light chain)	C
93.1	7MJL	ELECTRON MICROSCOPY	2.95	Alpha (N501Y)	A	Synthetic, Ab (heavy chain)	B
						Synthetic, Ab (Light chain)	C
94.1	7ND6	ELECTRON MICROSCOPY	7.3	Wh	B	Homo sapiens, Ab (heavy chain)	D
						Homo sapiens, Ab (Light chain)	E
95.1	7NEG	X-RAY DIFFRACTION	2.19	Alpha (N501Y)	C	Homo sapiens, Ab (heavy chain)	A
						Homo sapiens, Ab (Light chain)	B
96.1	7NEH	X-RAY DIFFRACTION	1.77	Wh	C	Homo sapiens, Ab (heavy chain)	A
						Homo sapiens, Ab (Light chain)	B
97.1	7NX7	X-RAY DIFFRACTION	2.3	Bata (K417N)	C	Homo sapiens, Ab (heavy chain)	D
						Homo sapiens, Ab (Light chain)	E
98.1	7NX8	X-RAY DIFFRACTION	1.95	Gamma	C	Homo sapiens, Ab (heavy chain)	D
						Homo sapiens, Ab (Light chain)	E
99.1	7NX9	X-RAY DIFFRACTION	2.4	Alpha	C	Homo sapiens, Ab (heavy chain)	D
						Homo sapiens, Ab (Light chain)	E
100.1	7NXA	X-RAY DIFFRACTION	2.5	Beta	C	Homo sapiens, Ab (heavy chain)	D
						Homo sapiens, Ab (Light chain)	E
101.1	7NXB	X-RAY DIFFRACTION	2.67	Gamma	C	Homo sapiens, Ab (heavy chain)	D
						Homo sapiens, Ab (Light chain)	E
102.1	7OAU	X-RAY DIFFRACTION	1.65	Alpha	A	Lama glama, Nanobody	B
103.1	7ORA	X-RAY DIFFRACTION	2.6	Delta (T478K)	A	Homo sapiens, Ab (heavy chain)	D
						Homo sapiens, Ab (Light chain)	E
104.1	7ORB	X-RAY DIFFRACTION	2.5	Delta (L452R)	A	Homo sapiens, Ab (heavy chain)	B
						Homo sapiens, Ab (Light chain)	C
104.2	7ORB	X-RAY DIFFRACTION	2.5	Delta (L452R)	A	Homo sapiens, Ab (heavy chain)	D
						Homo sapiens, Ab (Light chain)	E

Note: xxx.1 refers to the Nb that interacts with the RBM of RBD, while xxx.2 and xxx.3 denotes the Nb that interacts with other domains of RBD.
