# Supplementary material for: Computational design of novel nanobodies targeting the receptor binding domain of variants of concern of SARS-CoV-2
Source: PLoS One. 2023 Oct 24;18(10):e0293263. doi: 10.1371/journal.pone.0293263 (PMC10597523; doi:10.1371/journal.pone.0293263)
Supplement: S2 Table — (PDF) [file pone.0293263.s002.pdf]

S2 Table.

RBD strain	PDB ID	Structure investigation method	Resolution (Å)	Chain
Wh	7MFU	X-RAY DIFFRACTION	1.7	A
Alpha	7MJH	ELECTRON MICROSCOPY	2.66	A
Beta	7WD8	ELECTRON MICROSCOPY	4.30	A
Delta	7W9E	ELECTRON MICROSCOPY	3.10	A
Gamma	7NXB	X-RAY DIFFRACTION	2.67	C
Omicron BA.1	7XO5	ELECTRON MICROSCOPY	3.13	A
Omicron BA.2	7UB0	ELECTRON MICROSCOPY	3.31	A
