# Supplementary material for: Computational design of novel nanobodies targeting the receptor binding domain of variants of concern of SARS-CoV-2
Source: PLoS One. 2023 Oct 24;18(10):e0293263. doi: 10.1371/journal.pone.0293263 (PMC10597523; doi:10.1371/journal.pone.0293263)
Supplement: S3 Table — (PDF) [file pone.0293263.s003.pdf]

S3 Table.

Name	PDB ID	Structure investigation method	Resolution (Å)	Chain
ACE2	6VW1	X-RAY DIFFRACTION	2.68	A
Sars-CoV-1 RBD	2GHV	X-RAY DIFFRACTION	2.20	A
MERS RBD	6C6Y	X-RAY DIFFRACTION	3.32	E
HCoV-229E RBD	7CYC	ELECTRON MICROSCOPY	3.21	A
HCoV-NL63 RBD	3KBH	X-RAY DIFFRACTION	3.31	B
HCoV-HKU1 RBD	5KWB	X-RAY DIFFRACTION	1.91	A
HCoV-OC43 HA	6NZK	ELECTRON MICROSCOPY	2.80	A
H3N2 HA	4WE4	X-RAY DIFFRACTION	2.35	A
H1N1 HA	3MLH	X-RAY DIFFRACTION	2.09	A
