# Supplementary material for: Computational design of novel nanobodies targeting the receptor binding domain of variants of concern of SARS-CoV-2
Source: PLoS One. 2023 Oct 24;18(10):e0293263. doi: 10.1371/journal.pone.0293263 (PMC10597523; doi:10.1371/journal.pone.0293263)
Supplement: S4 Table — (PDF) [file pone.0293263.s004.pdf]

S4 Table.

	**Nb17.1**	**Nb17.1_Wh(8)**	**Nb17.1_A(7)**	**Nb17.1_B(8)**	**Nb17.1_D(8)**	**Nb17.1_G(8)**	**Nb17.1_BA. 1(8)**	**Nb17.1_BA.2(8)**
Number of amino acids	119	119	119	119	119	119	119	119
Formula	$C_{589}H_{902}N_{164}O_{171}S_4$	$C_{625}H_{934}N_{174}O_{167}S_5$	$C_{628}H_{923}N_{167}O_{169}S_4$	$C_{620}H_{924}N_{170}O_{171}S_5$	$C_{623}H_{927}N_{171}O_{172}S_4$	$C_{617}H_{922}N_{172}O_{169}S_5$	$C_{633}H_{926}N_{172}O_{167}S_4$	$C_{613}H_{903}N_{167}O_{175}S_4$
Molecular weight	13144.88	13717.66	13644.49	13655.49	13692.5	13613.46	13745.61	13540.17
Ext. coefficient at OD280 (M^{-1} cm^{-1})	39545	57535	64525	53525	55015	52035	68535	56045
