# Supplementary material for: Computational design of novel nanobodies targeting the receptor binding domain of variants of concern of SARS-CoV-2
Source: PLoS One. 2023 Oct 24;18(10):e0293263. doi: 10.1371/journal.pone.0293263 (PMC10597523; doi:10.1371/journal.pone.0293263)
Supplement: S5 Table — (PDF) [file pone.0293263.s005.pdf]

**S5 Table.**

| | **Nb23.1** | **Nb23.1_Wh(8)** | **Nb23.1_A(6)** | **Nb23.1_B(8)** | **Nb23.1_D(7)** | **Nb23.1_G(8)** | **Nb23.1_BA. 1(8)** | **Nb23.1_BA.2(6)** |
|---|---|---|---|---|---|---|---|---|
| Number of amino acids | 115 | 115 | 115 | 115 | 115 | 115 | 115 | 115 |
| Formula | $C_{556}H_{859}N_{151}O_{172}S_4$ | $C_{579}H_{889}N_{159}O_{170}S_5$ | $C_{576}H_{873}N_{155}O_{173}S_5$ | $C_{585}H_{886}N_{162}O_{170}S_4$ | $C_{576}H_{875}N_{155}O_{171}S_5$ | $C_{583}H_{892}N_{160}O_{169}S_5$ | $C_{586}H_{885}N_{157}O_{169}S_5$ | $C_{568}H_{878}N_{160}O_{170}S_4$ |
| Molecular weight | 12539.09 | 12957.69 | 12897.50 | 13036.69 | 12867.52 | 13006.77 | 12993.72 | 12796.43 |
| Ext. coefficient at OD280 ($M^{-1}$ $cm^{-1}$) | 26025 | 33015 | 33015 | 38515 | 37025 | 34505 | 35995 | 26025 |
