# Supplementary figures and images for: Computational design of novel nanobodies targeting the receptor binding domain of variants of concern of SARS-CoV-2
Source: PLoS One. 2023 Oct 24;18(10):e0293263. doi: 10.1371/journal.pone.0293263 (PMC10597523; doi:10.1371/journal.pone.0293263)

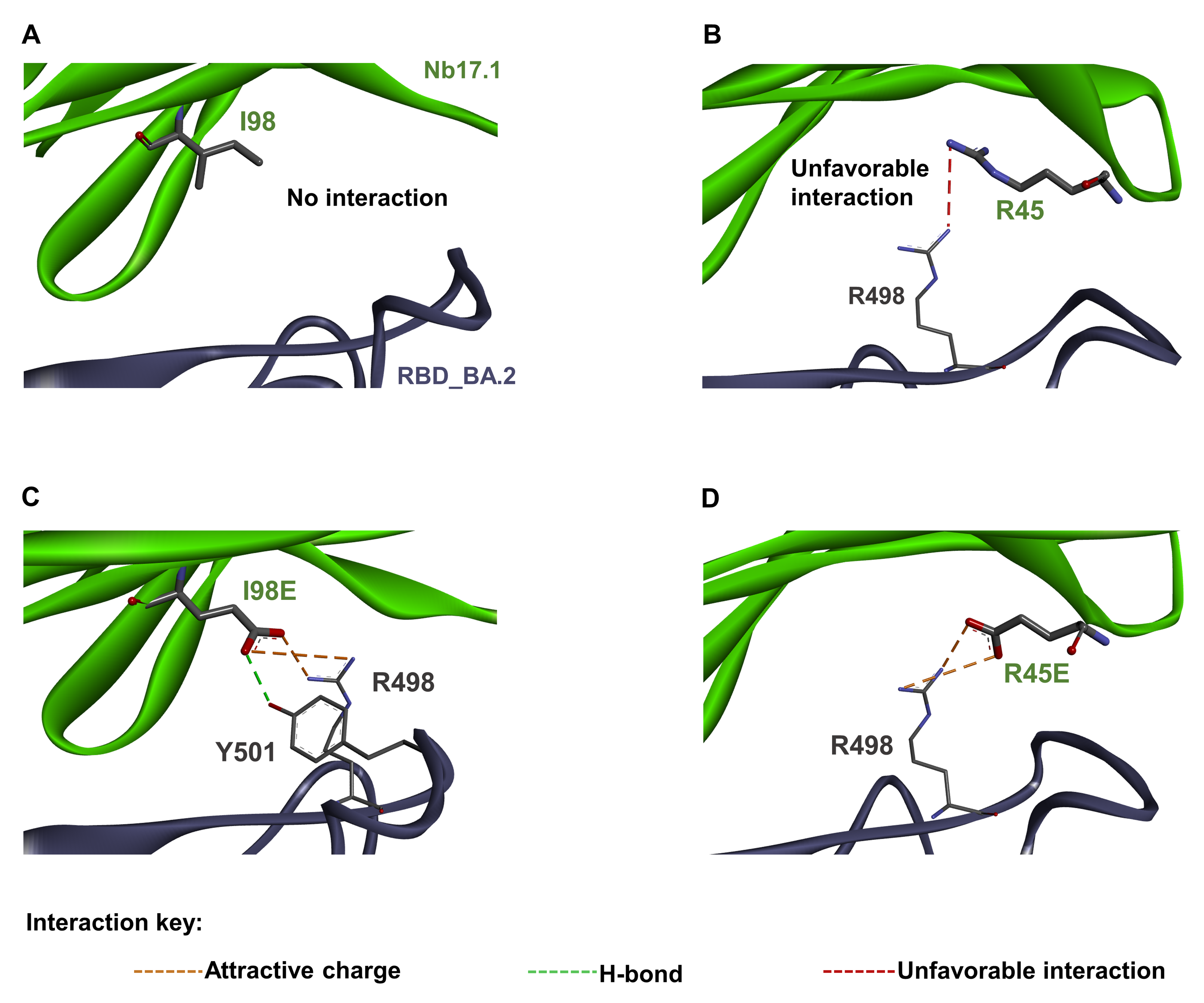

Supplement: S1 Fig — (A) representing a non-interacting residue, (B) an unfavorable interaction, and (C-D) the protein-protein interaction after mutation. (TIF) [file pone.0293263.s006.tif]

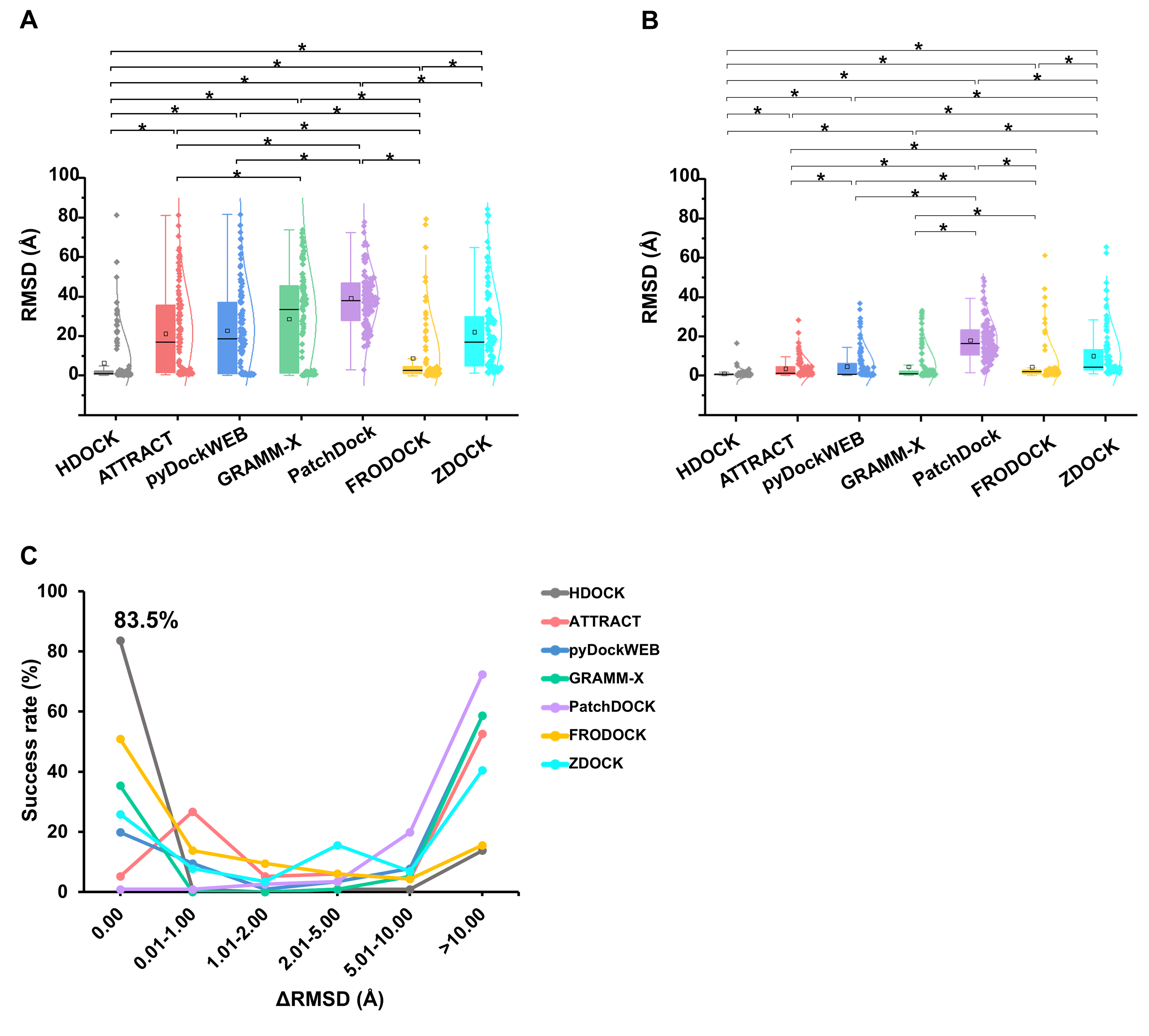

Supplement: S2 Fig — The RMSD distribution of (A) the top pose and (B) the best pose, where the asterisk indicates a significant difference (p < 0.05, Kruskal-Wallis with Dunn’s test) between programs. (C) The success of docking programs. (TIF) [file pone.0293263.s007.tif]

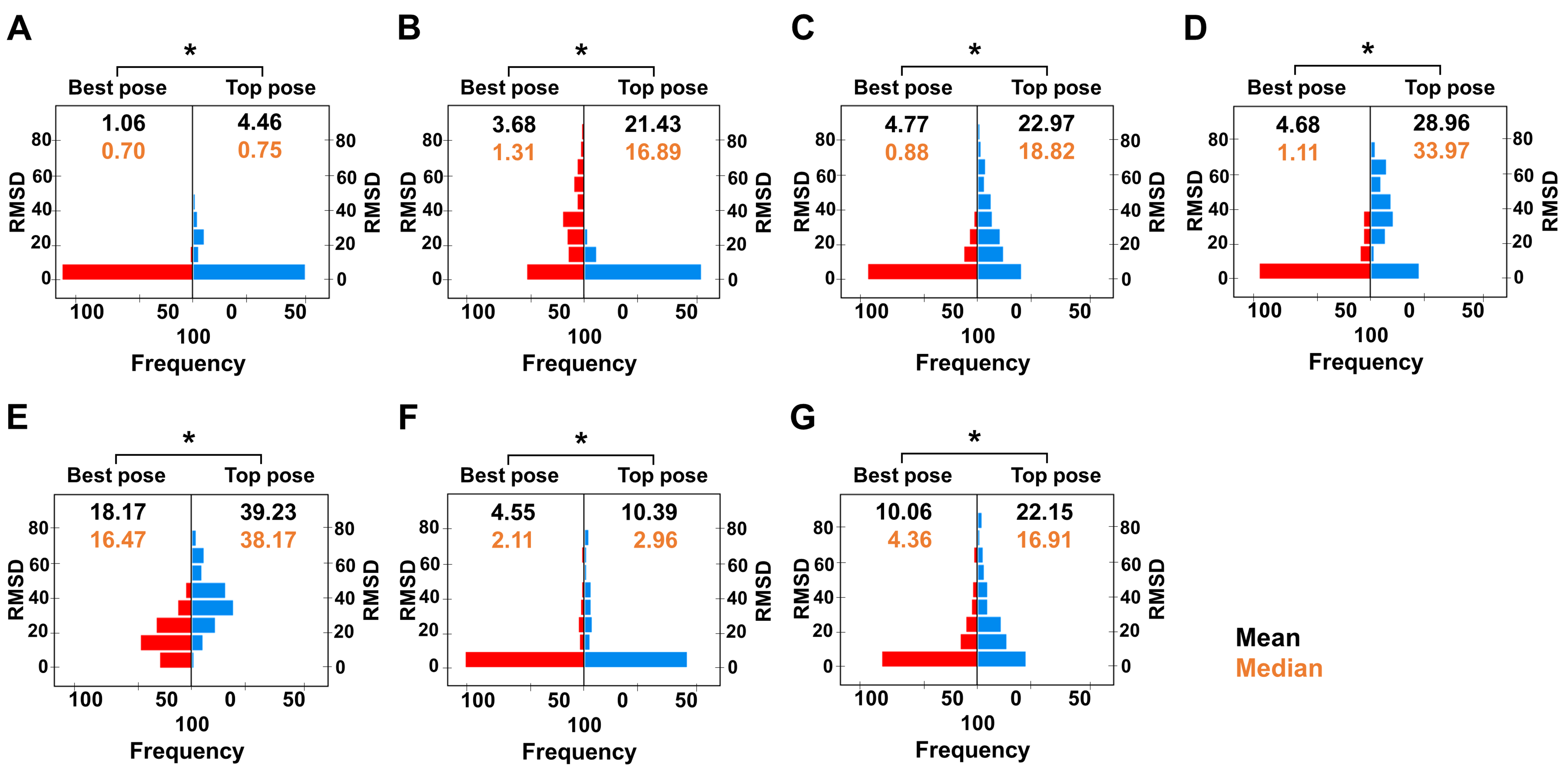

Supplement: S3 Fig — (A) HDOCK, (B) ATTRACT, (C) pyDockWEB, (D) GRAMM-X, (E) PatchDock, (F) FRODOCK, and (G) ZDOCK, for Nb/RBD and Ab/RBD complexes. The statistical significance was evaluated using a paired-sample Wilcoxon signed-rank test (* denotes significance at p < 0.05, N = 115). (TIF) [file pone.0293263.s008.tif]

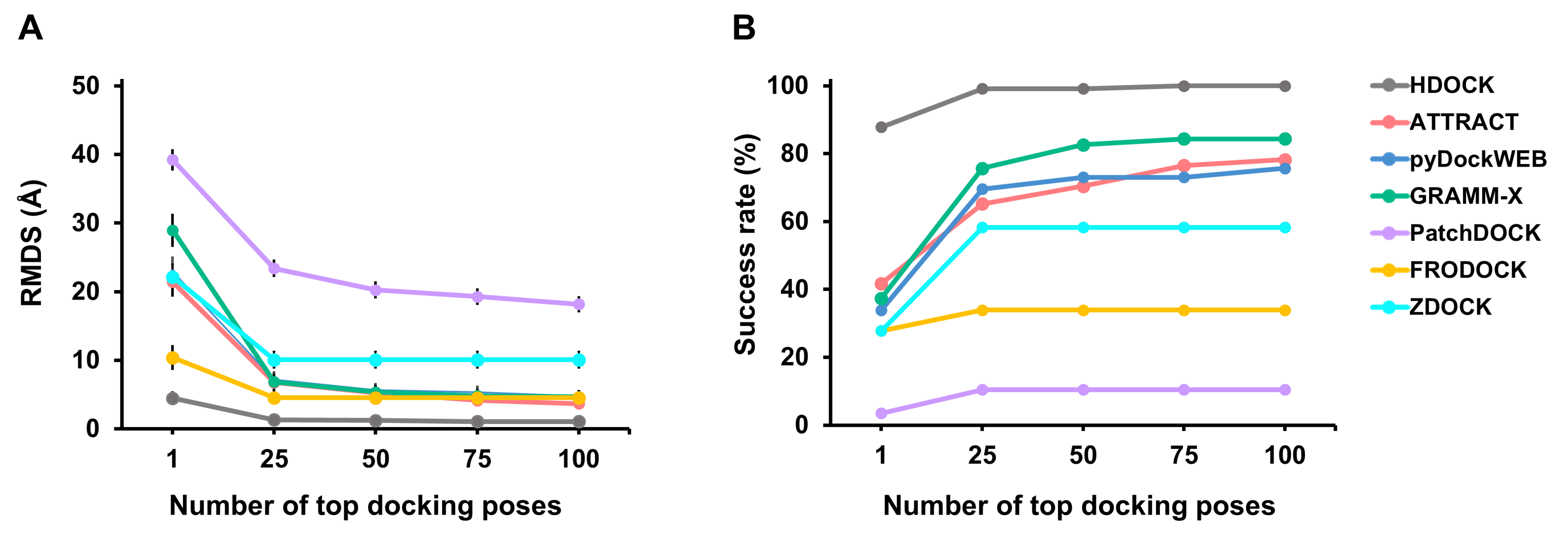

Supplement: S4 Fig — (A) the mean RMSD ± SEM and (B) success rate. Blind docking was performed on Nb/RBD and Ab/RBD datasets, with N = 115. (TIF) [file pone.0293263.s009.tif]

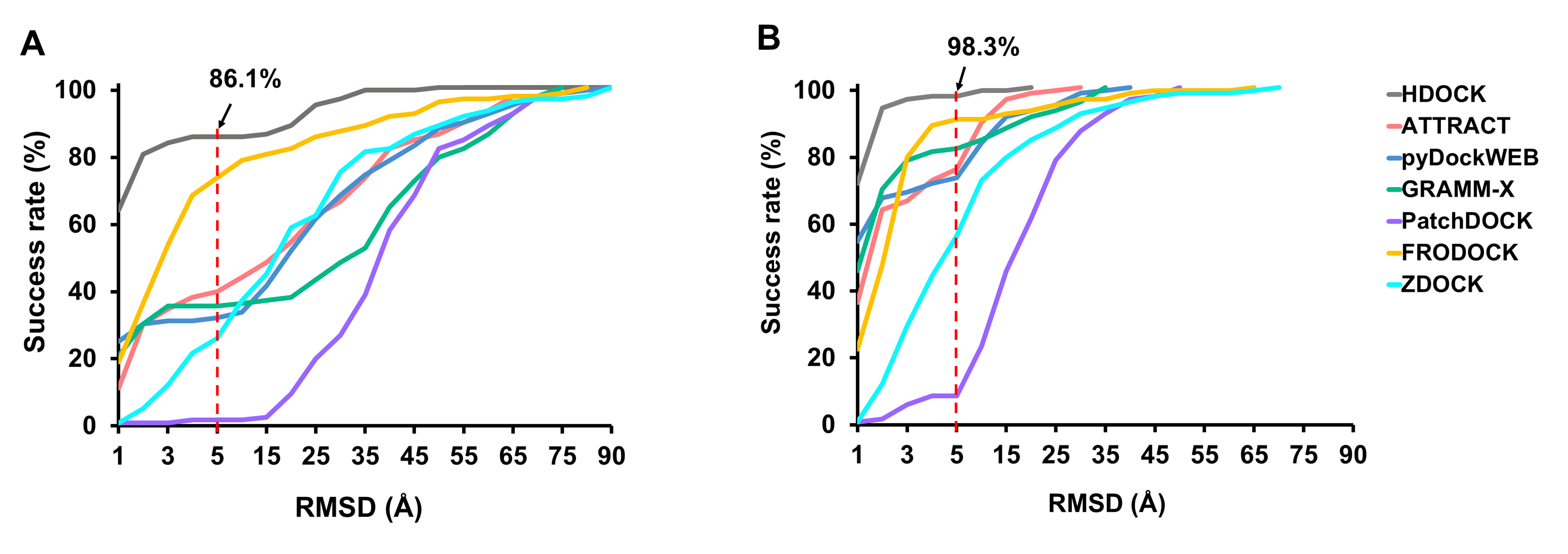

Supplement: S5 Fig — (A) top pose and (B) best pose obtained by different docking methods for Nb/RBD and Ab/RBD complexes, with N = 115. (TIF) [file pone.0293263.s010.tif]

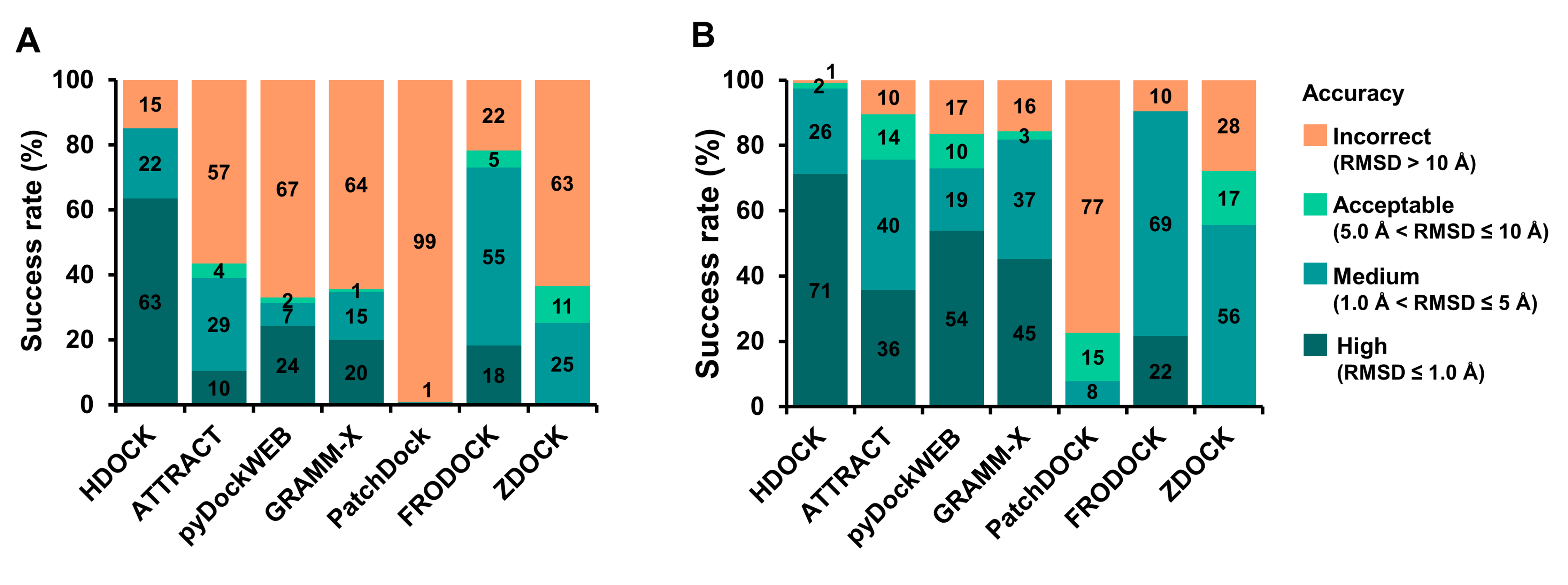

Supplement: S6 Fig — (A) the top pose RMSD and (B) the best pose RMSD were considered. (TIF) [file pone.0293263.s011.tif]

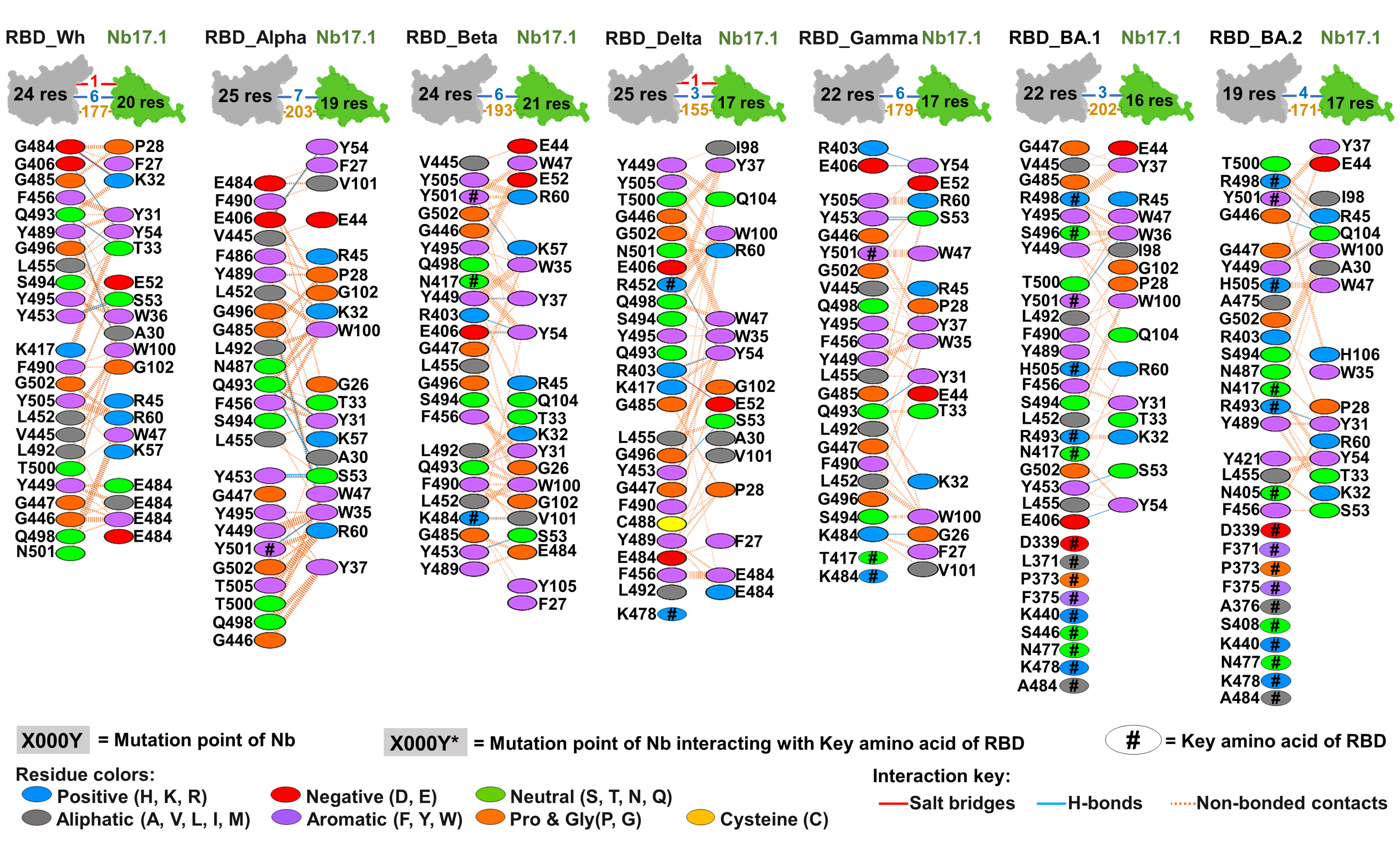

Supplement: S7 Fig — (TIF) [file pone.0293263.s012.tif]

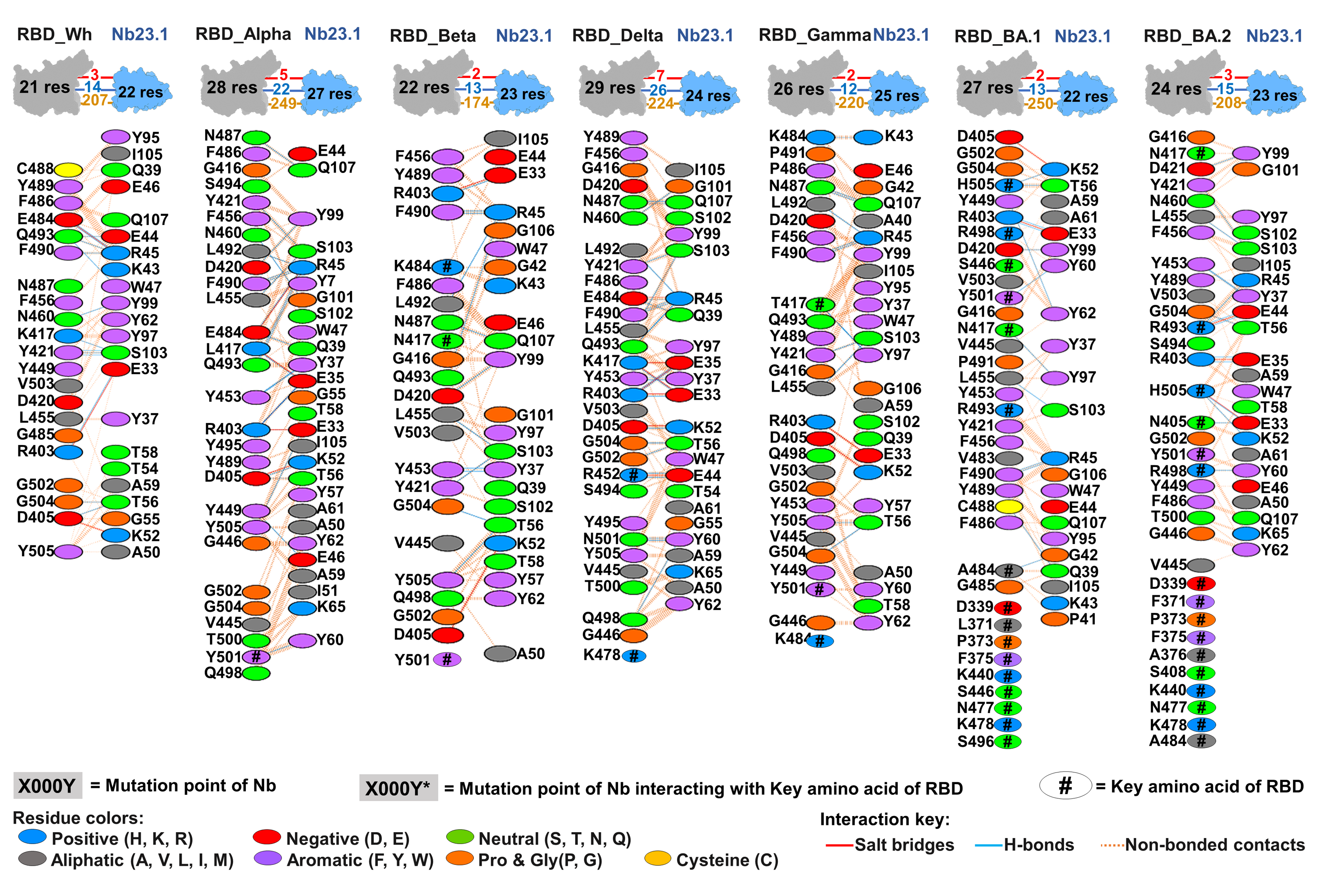

Supplement: S8 Fig — (TIF) [file pone.0293263.s013.tif]

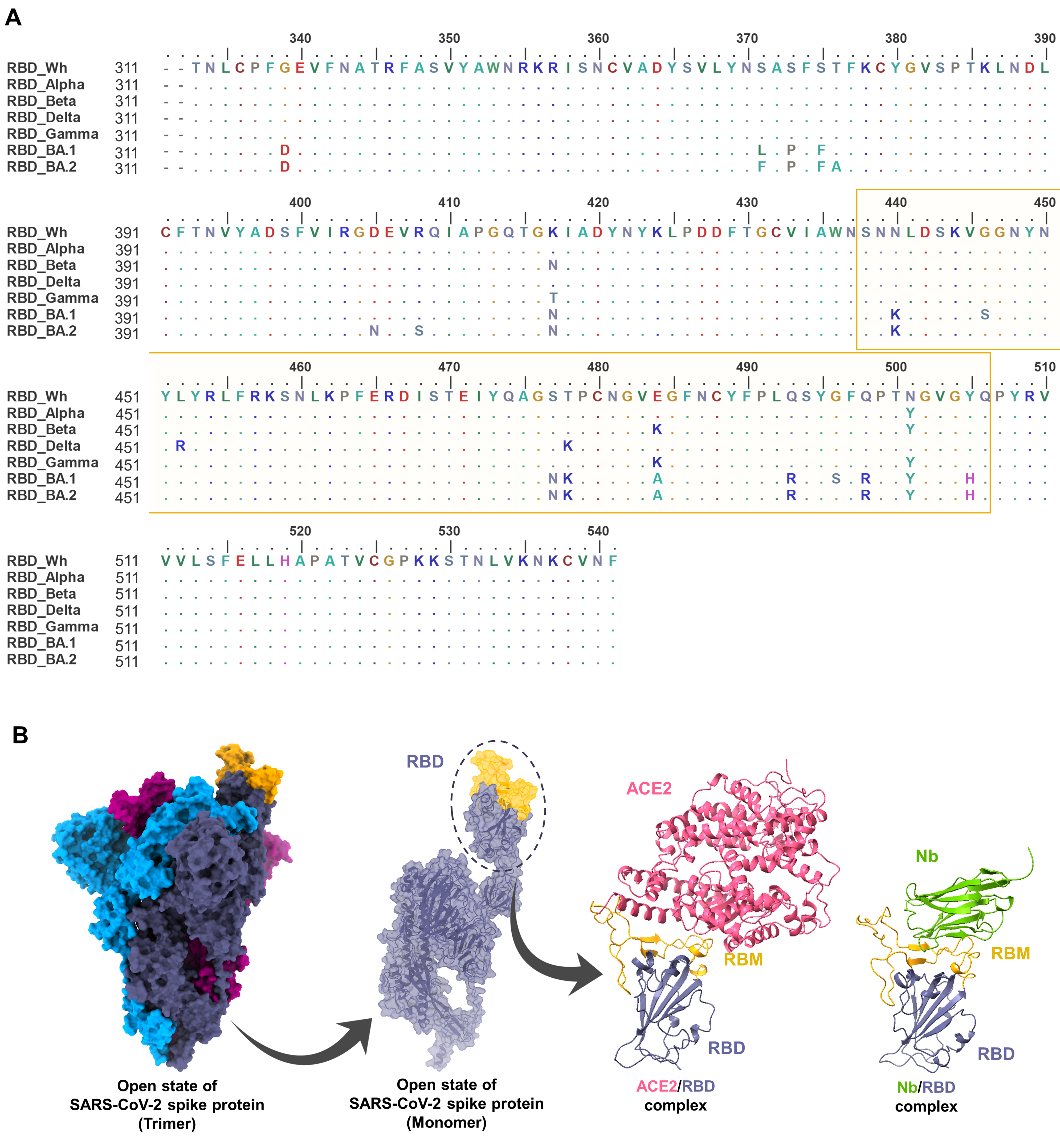

Supplement: S9 Fig — (A) The sequence alignment of SARS-CoV-2 RBDs and the comparison of RBM sequence in yellow border, and (B) interaction of ACE2/SARS-CoV-2 RBD and Nb/SARS-CoV-2 RBD. (TIF) [file pone.0293263.s014.tif]

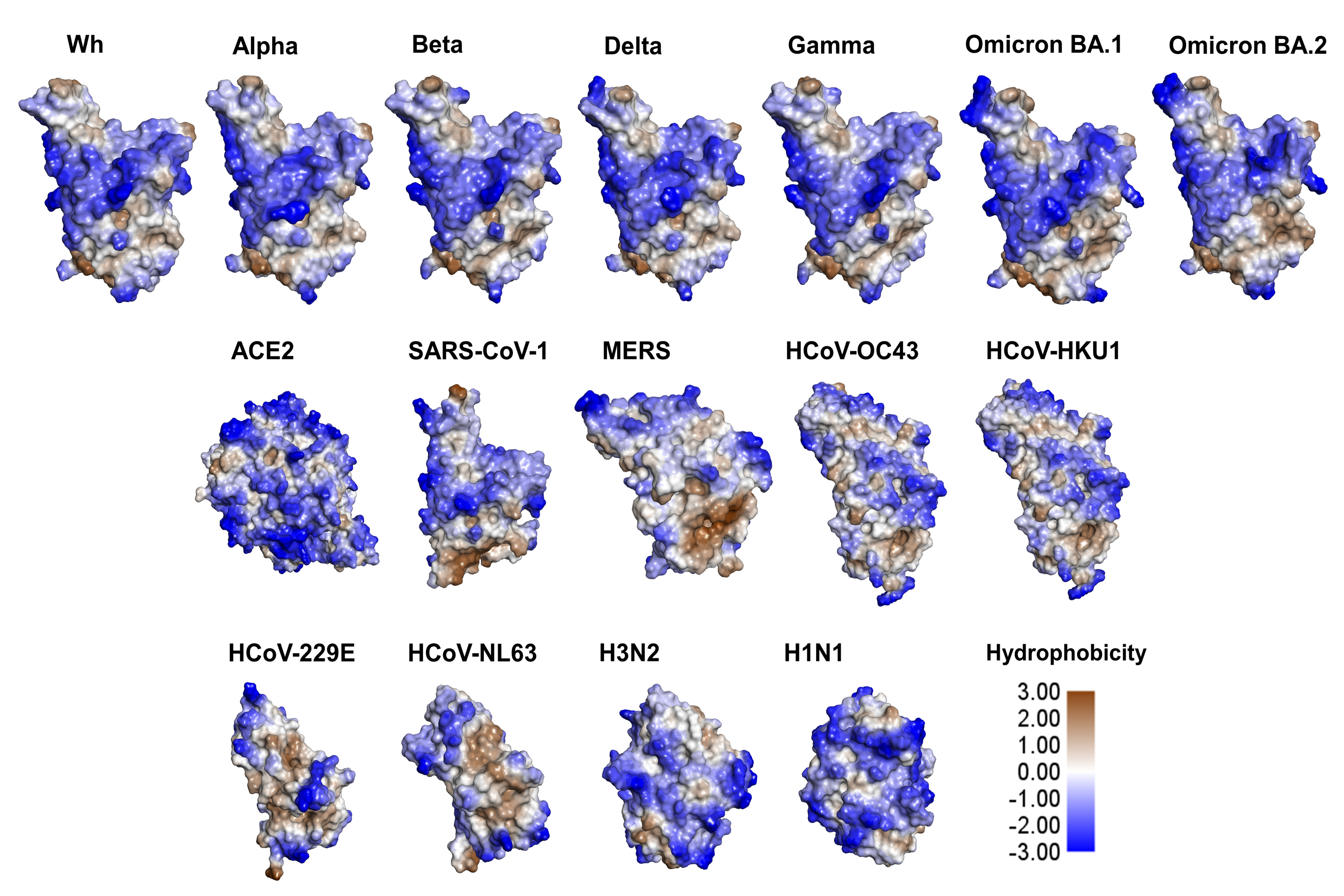

Supplement: S10 Fig — (TIF) [file pone.0293263.s015.tif]

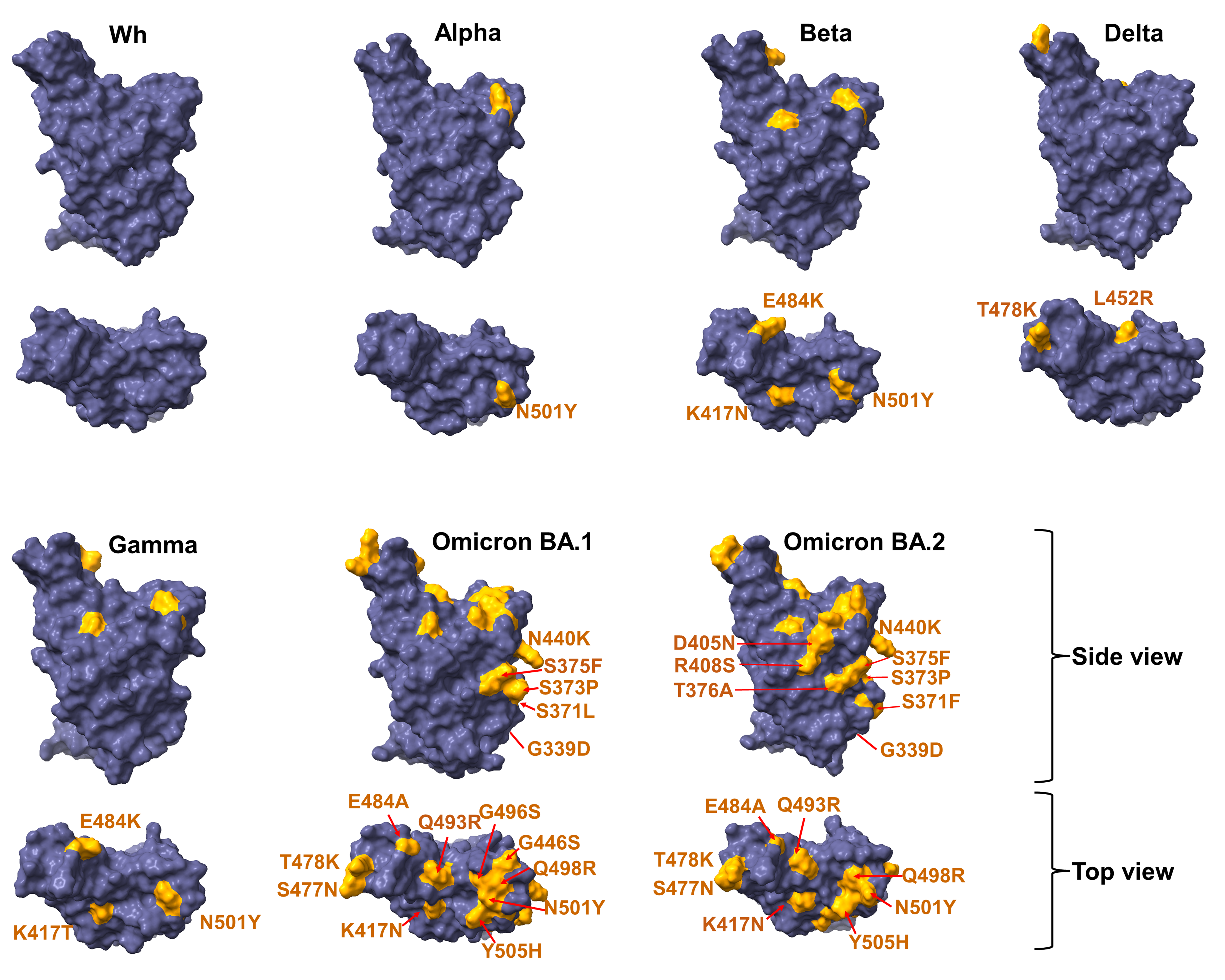

Supplement: S11 Fig — (TIF) [file pone.0293263.s016.tif]

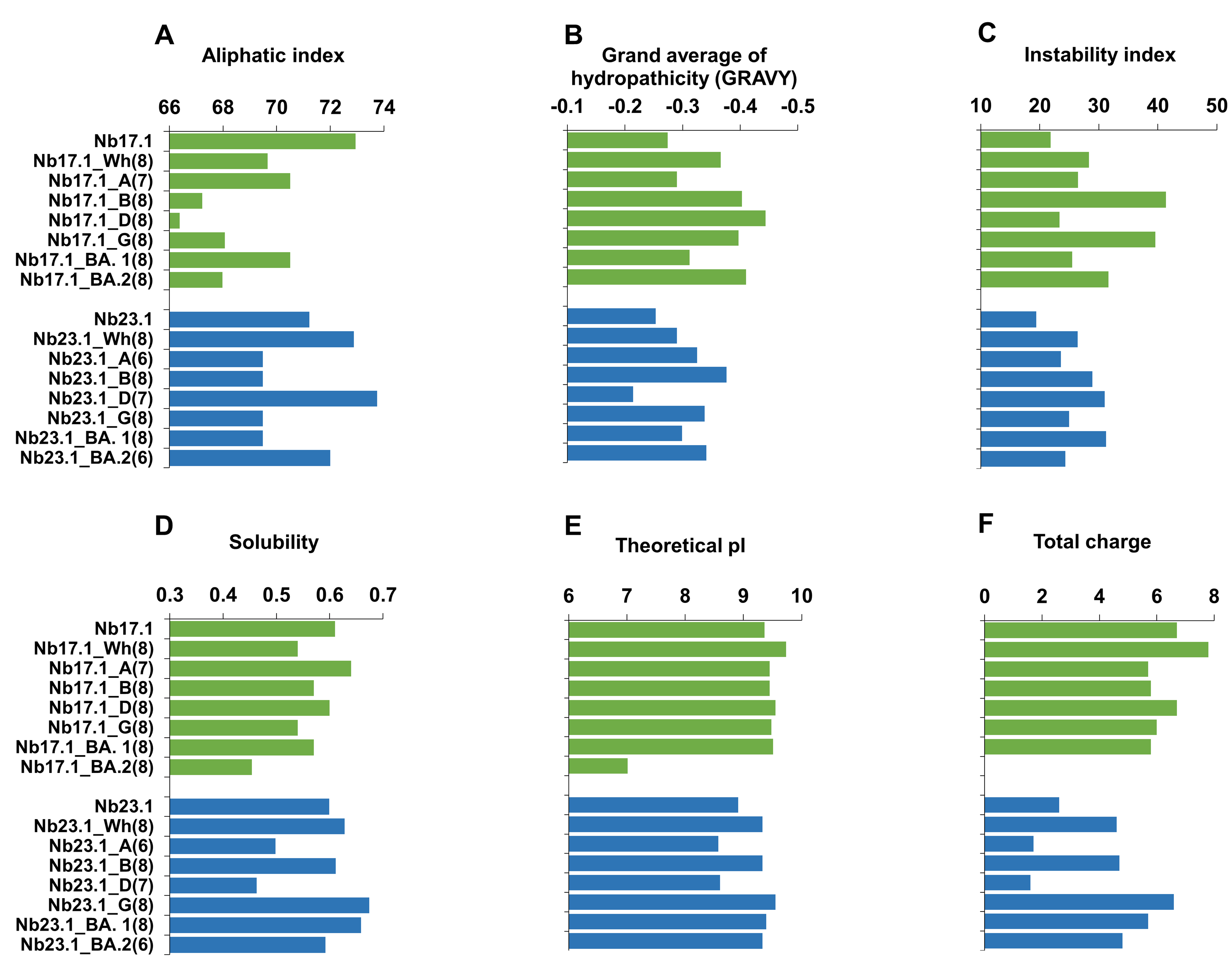

Supplement: S12 Fig — (A) aliphatic index, (B) grand average of hydropathicity (GRAVY), (C) instability index, (D) theoretical pI, (E) solubility, and (F) total charge of engineered (green bar) Nb17.1 and (blue bar) Nb23.1. (TIF) [file pone.0293263.s017.tif]

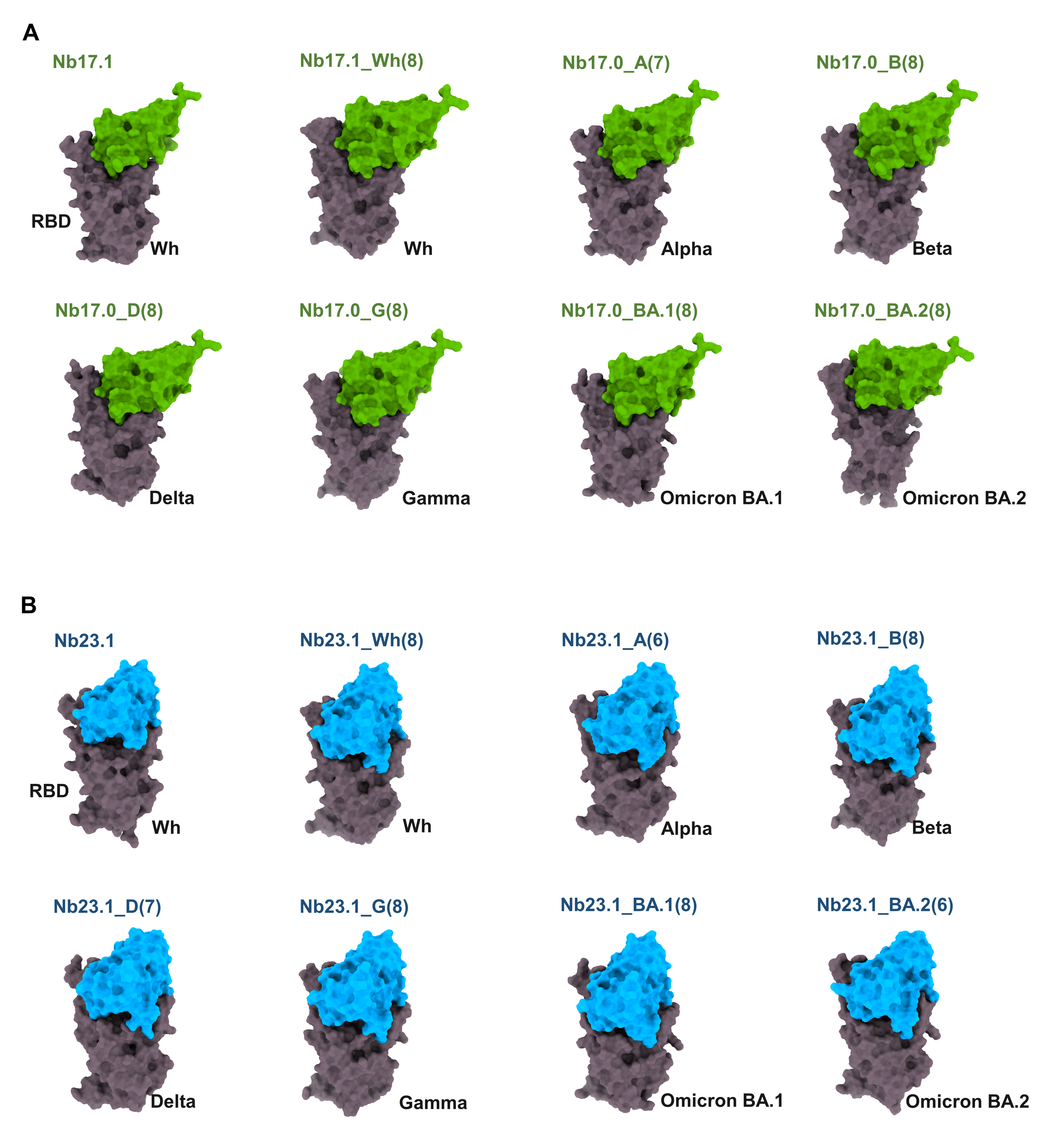

Supplement: S13 Fig — (A) Nb17.1 and (B) Nb23.1 with different targeted RBDs. (TIF) [file pone.0293263.s018.tif]
