# Supplementary material for: Computational design of novel nanobodies targeting the receptor binding domain of variants of concern of SARS-CoV-2
Source: PLoS One. 2023 Oct 24;18(10):e0293263. doi: 10.1371/journal.pone.0293263 (PMC10597523; doi:10.1371/journal.pone.0293263)
Supplement: Attachment — Submitted filename: Response to Reviewers.docx [file pone.0293263.s019.docx]

**RESPONSE TO REVIEWER’S COMMENTS**

**Ms. Ref. No.:** PONE-D-23-16784

**Title:** Computational design of novel nanobodies targeting the receptor binding domain of variants of concern of SARS-CoV-2

**Journal Requirements:**

1. Please ensure that your manuscript meets PLOS ONE's style requirements, including those for file naming.

**Responses:** We have followed the given instructions, and the revised information has appeared within the document labeled as 'manuscript'

2. Please note that PLOS ONE has specific guidelines on code sharing for submissions in which author-generated code underpins the findings in the manuscript. In these cases, all author-generated code must be made available without restrictions upon publication of the work. Please review our guidelines at https://journals.plos.org/plosone/s/materials-and-software-sharing#loc-sharing-code and ensure that your code is shared in a way that follows best practice and facilitates reproducibility and reuse.

**Responses:** We did not generate codes for execution as all procedures have been performed on free servers; we have included a link to the server for each program in the methods section of the manuscript.

3. We note that you have provided additional information within the Acknowledgements Section that is not currently declared in your Funding Statement. Please note that funding information should not appear in the Acknowledgments section or other areas of your manuscript. We will only publish funding information present in the Funding Statement section of the online submission form.

Please remove any funding-related text from the manuscript and let us know how you would like to update your Funding Statement.

**Responses:** The funding-related text was removed from the 'manuscript'.

The new acknowledgement statement reads as follows:

“P.L. would like to thank the Petchra Pra Jom Klao Ph.D. Research Scholarship from King Mongkut’s University of Technology Thonburi. We extend our gratitude to Dr. Nitchakan Darai and Miss Hathaichanok Chuntakaruk (Program in Bioinformatics and Computational Biology, Graduate School, Chulalongkorn University) for their insightful discussions and technical assistance.”

*According to the university's regulations, including the message 'the Petchra Pra Jom Klao Ph.D. Research Scholarship from King Mongkut’s University of Technology Thonburi' in the acknowledgment section is required.

Please include your amended statements within your cover letter; we will change the online submission form on your behalf.

**Responses:** The funding statement has been modified and included in the cover letter. Currently, our Funding Statement reads as follows:

“Research project is supported by the National Research Council of Thailand (NRCT) and King Mongkut’s University of Technology Thonburi: N42A650316, the Research Strengthening Project of the Faculty of Engineering and Thailand Science Research and Innovation (TSRI), Basic Research Fund: Fiscal year 2023 (Program Smart Healthcare). P.L. gratefully acknowledge the financial support provided by the Petchra Pra Jom Klao Ph.D. Research Scholarship from King Mongkut’s University of Technology Thonburi. T.R. is financially supported by the National Research Council of Thailand (NRCT, grant number N42A650231).”

4. In your Data Availability statement, you have not specified where the minimal data set underlying the results described in your manuscript can be found. PLOS defines a study's minimal data set as the underlying data used to reach the conclusions drawn in the manuscript and any additional data required to replicate the reported study findings in their entirety. All PLOS journals require that the minimal data set be made fully available. For more information about our data policy, please see <http://journals.plos.org/plosone/s/data-availability>.

Upon re-submitting your revised manuscript, please upload your study’s minimal underlying data set as either Supporting Information files or to a stable, public repository and include the relevant URLs, DOIs, or accession numbers within your revised cover letter. For a list of acceptable repositories, please see <http://journals.plos.org/plosone/s/data-availability#loc-recommended-repositories>. Any potentially identifying patient information must be fully anonymized.

**Responses:** In the manuscript, the validation dataset's dimensions are detailed on line 74 (consisting of 115 nanobodies and antibodies). Additionally, the S1-S3 table containing all relevant PDB IDs is provided. Regarding the study findings, we've included a sample PDB file (<https://osf.io/fesr8/?view_only=c38b2ebe17ce4c81997c5418a7fe7e84>) showcasing the altered Nb structure for your review.

5. Please upload a new copy of all Figures as the details are not clear.

**Responses:** We have followed the provided instructions to handle the images and have successfully uploaded all the files.

6. PLOS requires an ORCID iD for the corresponding author in Editorial Manager on papers submitted after December 6th, 2016. Please ensure that you have an ORCID iD and that it is validated in Editorial Manager. To do this, go to ‘Update my Information’ (in the upper left-hand corner of the main menu), and click on the Fetch/Validate link next to the ORCID field. This will take you to the ORCID site and allow you to create a new iD or authenticate a pre-existing iD in Editorial Manager. Please see the following video for instructions on linking an ORCID iD to your Editorial Manager account: <https://www.youtube.com/watch?v=_xcclfuvtxQ>

**Responses:** We have successfully comprehended and implemented the instructions.

Rungtiva P. Poo-arporn; <https://orcid.org/0000-0002-2584-9890>

Thanyada Rungrotmongkol; <https://orcid.org/0000-0002-7402-3235>

**Comments to the Author:**

1. Is the manuscript technically sound, and do the data support the conclusions?

The manuscript must describe a technically sound piece of scientific research with data that supports the conclusions. Experiments must have been conducted rigorously, with appropriate controls, replication, and sample sizes. The conclusions must be drawn appropriately based on the data presented.

**Reviewer #1:** Partly

**Responses:** We have submitted the figure along with the essential details for your review.

2. Has the statistical analysis been performed appropriately and rigorously?

**Reviewer #1:** N/A

**Responses:** The statistical analyses were conducted as depicted in the 'manuscript', and the outcomes of these tests are presented in the attached experimental result figures.

3. Have the authors made all data underlying the findings in their manuscript fully available?

The PLOS Data policy requires authors to make all data underlying the findings described in their manuscript fully available without restriction, with rare exception (please refer to the Data Availability Statement in the manuscript PDF file). The data should be provided as part of the manuscript or its supporting information or deposited to a public repository. For example, in addition to summary statistics, the data points behind means, medians and variance measures should be available. If there are restrictions on publicly sharing data—e.g. participant privacy or use of data from a third party—those must be specified.

**Reviewer #1:** No

**Responses:** We have included study results data in the form of images, diagrams, and tables, along with attached statistical analysis data, all provided for your consideration.

4. Is the manuscript presented in an intelligible fashion and written in standard English?

PLOS ONE does not copyedit accepted manuscripts, so the language in submitted articles must be clear, correct, and unambiguous. Any typographical or grammatical errors should be corrected at revision, so please note any specific errors here.

**Reviewer #1:** No

**Responses:** We have undertaken a review to ensure language precision and to identify potential typographical or grammatical errors.

5. Review Comments to the Author

Please use the space provided to explain your answers to the questions above. You may also include additional comments for the author, including concerns about dual publication, research ethics, or publication ethics. (Please upload your review as an attachment if it exceeds 20,000 characters)

**Reviewer #1:** All main figures are missing and are not included in the manuscript file for review. Without the complete data/figures available, the reviewer cannot make a sound judgement on the quality of the manuscript.

**Responses:** Thank you for your precious time to review our manuscript as we submit the manuscript and Figure within the PLOS ONE criteria as the instruction said

“Please remove your figures from within your manuscript file, leaving only the individual TIFF/EPS image files.  These will be automatically included in the reviewer’s PDF” The figures are all available for download after the text.

However, we’ve now uploaded images, test results, and essential information for your review and we look forward to being reconsidered. They are also available via the provided link below.

<https://osf.io/fesr8/?view_only=c38b2ebe17ce4c81997c5418a7fe7e84>

<https://figshare.com/s/070fed60428a4d6ca20b>
